# An integrated subtractive genomics and immunoinformatic approach for designing a multi-epitope peptide vaccine against methicillin-resistant *Staphylococcus aureus*

**DOI:** 10.3389/fbinf.2025.1745495

**Published:** 2026-01-14

**Authors:** Nandha Kumar Subramani, Subhashree Venugopal, Anand Prem Rajan

**Affiliations:** 1 Department of Bio Medical Sciences, School of Bio Science and Technology, Vellore Institute of Technology, Vellore, Tamil Nadu, India; 2 Department of Integrative Biology, School of Bio Science and Technology, Vellore Institute of Technology, Vellore, Tamil Nadu, India

**Keywords:** immune simulation, molecular dynamicsimulation, MRSA, multi-epitope vaccine, subtractive genomic method

## Abstract

**Introduction:**

MRSA is a multi-drug-resistant bacteria responsible for severe infections that has become a major health concern. Due to constraints of traditional methods, there is a need for developing a new approach to prevent the MRSA-related infections by targeting key pathogens.

**Methods:**

Initially, the subtractive genomics was applied to the MRSA proteome to identify non-homologous, essential, and virulence targets using comparative BLAST-based screening. Further, immunoinformatic tools were employed for B- and T-cell epitope prediction and vaccine construction with appropriate adjuvants and linkers, followed by immune simulation and molecular docking with immune receptors.

**Results:**

Comparative metabolic pathway analysis identified 294 MRSA pathway proteins, with acetolactate synthase (ALS) as a non-homologous, essential, and virulent protein that is involved in the branched amino acid biosynthesis pathway. The constructed ALS vaccine consists of 3 B-cell and 19 T-cell epitopes exhibited stable immunological features with 97.55% global population coverage. Molecular docking revealed that ALS exhibited a superior binding affinity with the TLR4 receptor (−1,438.7 kcal/mol) than the TLR2 receptor (−1,103.5 kcal/mol), which was further confirmed by high structural stability and compactness analysis. Immune simulations also exhibited elevated IgM, IgG subtypes, and cytokine productions, suggesting a robust humoral and cellular immunity.

**Discussion:**

Identified ALS highlights its biological relevance in MRSA survival. The stability predictions with TLR4 suggested effective activation of innate immunity that may enhance antigen presentation and downstream adaptive immunity. The validation of the ALS vaccine’s safety and immunogenicity further requires comprehensive *in vitro* and *in vivo* examinations.

**Conclusion:**

Thus, ALS is recognized as a promising MRSA vaccine candidate and has the potential to activate immune responses effectively.

## 1 Introduction

The consistent emergence and spread of antimicrobial resistance (AMR) have become major health concerns, as the resistant pathogen causes a wide range of community-associated and hospital-associated infections. When combating disease-associated pathogens, AMR remains a complex problem, which requires a lot of attention. One such important AMR pathogen is methicillin-resistant *Staphylococcus aureus* (MRSA). It is a non-motile, non-sporulating, catalase-positive, facultative anaerobic, gram-positive coccus derived from the Staphylococcaceae family (Clebak and Malone, 2018). It is a deadly pathogen that causes nosocomial, healthcare, and community-associated illnesses. Recently, a meta-analysis study reported that MRSA global prevalence in 2023 was found to be 14.69% with a 95% confidence interval of 12.39%–17.15% (Hasanpour et al., 2023). Furthermore, the WHO reported that MRSA causes severe bloodstream infections in hospitalized individuals, with statistics reaching 32.2% in 2022.

In addition to that, MRSA is recognized for its broad range of pyogenic infections, especially impacting skin infections like staphylococcal scalded skin syndrome, folliculitis, cellulitis, impetigo, pneumonia, endocarditis, osteomyelitis, etc (Rønning et al., 2025; WHO, 2024). These infections are highly associated with higher morbidity and mortality rates than methicillin-susceptible strains. This contributes to prolonged hospitalization, complexity, and failure of therapeutic treatments. For instance, the recent studies projected the antimicrobial resistance caused by various microorganisms, including MRSA, will inflict economic deprivation up to $2 trillion per year by 2050 worldwide. This highlights public health concerns and economic requirements to advance preventive measures like vaccine development (Ayau et al., 2017; B. B. Gupta et al., 2021).

Subsequently, the secretion of virulent factors in MRSA is predominately responsible for immune evasion, colonization, biofilm formation, and tissue destruction. The list of predominant virulent factors that are resistant to commercial antibiotics includes adherence (clumping factor, collagen-binding protein, elastin-binding protein, and fibronectin-binding protein), exotoxins (α-hemolysin, β-hemolysin, γ-hemolysin, staphylococcal enterotoxin, staphylococcal superantigen-like protein, and toxic shock syndrome toxin-1), and exoenzymes (exfoliative toxin, hyaluronate lyase, staphylokinase, and staphylocoagulase) (De Jong et al., 2019; Deurenberg and Stobberingh, 2008; Wang et al., 2022).

The identification of relevant drug targets and effective antibiotics in combating MRSA remains a major issue. Primarily, the continuous reliance on existing drugs for MRSA makes them ineffective, thus leading to the development of resistance mechanisms. For instance, the therapeutic arsenal, such as vancomycin, a primary agent for MRSA, and alternative existing medicines such as telavancin, cefazoline, oxazolidinones, teicoplanin, and daptomycin, mainly focuses on traditional drug targets that are involved in cell wall synthesis and protein synthesis (Keikha and Karbalaei, 2024). Further, the emergence of resistant mutants examined in the higher dosages also leaves adverse side effects such as nephrotoxicity, peripheral neuropathy, myelosuppression, renal toxicity, and creatine phosphokinase elevation. Thus, introducing new alternative approaches effectively prevents the infections caused by MRSA through identifying key targets.

Screening key virulent proteins from existing data becomes crucial to inhibit the activity of MRSA in the host. These proteins are utilized for developing effective vaccines that stimulate immune responses in the host tissue and reduce reliance on anti-MRSA drug interventions. Considering this, many bioinformatics methods are developed to predict MRSA virulent proteins and prevent MRSA-associated infections, which include comparative genomics, reverse vaccinology, network pharmacology, and genome-wide analysis such as core genomic and subtractive genomic methods (Karim et al., 2020; Khan et al., 2022b; Lyon et al., 2025; Naorem et al., 2022; Zhai et al., 2025). Among these, the subtractive genomics approach plays a vital role in identifying potential virulence targets. It is widely used for identifying bacterial proteins that are non-homologous and essential for bacterial survival. Mostly, proteins are retrieved from metabolic pathways, whole genomes, or whole proteomes. In contrast, the reverse vaccinology approach is able to identify immunogenic antigens and prioritize vaccine candidates that are less susceptible to immune evasion and reduce adverse cross-reactivity, thereby enabling effective targeting of multidrug-resistant pathogens.

To accomplish that, this study utilized the subtractive genomic method to identify unique metabolic pathway (KEGG pathway database) proteins from the whole proteome to prioritize targets that are non-homologous (NCBI BLASTp) to humans, essential (DEG database) for MRSA survival, and virulent (VFDB and VICMpred database) for MRSA pathogenicity (Khan et al., 2022a). Following the identification essential targets, reverse vaccinology principles were used for designing multi-epitope vaccines, which are known to reduce the duration for vaccine discovery, toxicity prediction, and allergic reaction prediction (Kumar et al., 2024; Subramani and Venugopal, 2025). Afterwards, the constructed vaccine was docked against immune receptors, and its structural stabilities were examined using molecular dynamic simulations. Then, the immune response profile between the human immune system and the MRSA protein vaccine was employed through C-ImmSim (immune response simulation) software (Garrido-Palazuelos et al., 2024). Overall, this study focused on identifying novel MRSA virulent targets through subtractive genome analysis and constructing a multi-epitope vaccine for suppressing the activity of MRSA through immunoinformatic approaches.

## 2 Methods

**FIGURE 1:**
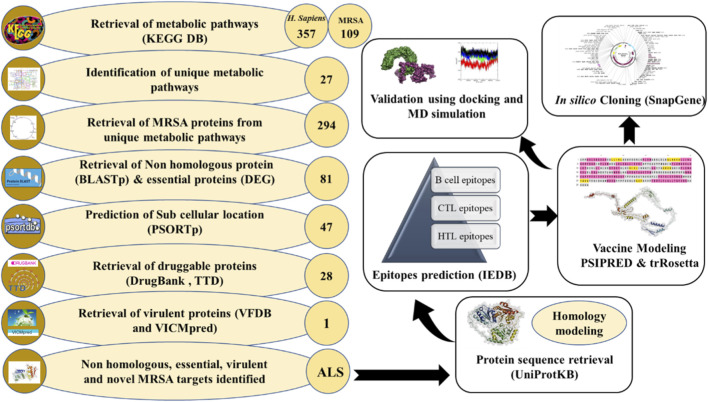
A schematic representation of the workflow used in selection of novel protein and developing a multi-epitope vaccine against MRSA.

This study contains two phases; phase I contains the subtractive genomic analysis for identification of novel proteins, and phase II contains multi-epitope vaccine constructions using an immunoinformatic approach. The Figure 1 illustrates the overall methodology for both Phase I and Phase II.

### 2.1 Subtractive genomic analysis

#### 2.1.1 Retrieval of unique metabolic pathways

The complete metabolic pathways of human and MRSA bacteria were obtained from the Kyoto Encyclopedia of Genes and Genomes (KEGG) database (Kanehisa et al., 2025). It’s a large repository containing manually curated and mapped metabolic pathways, molecular networks, and interactions for many species (https://www.kegg.jp/kegg/, last accessed July 2025). The pathway of MRSA (KEGG ID: T00182, Org code: sar) was manually compared with the metabolic pathways of *Homo sapiens* (KEGG ID: T01001, Org code: hsa) to identify unique and common pathways. Among them, only the unique metabolic pathways of MRSA were utilized for further analysis, which minimizes the potential cross interactions with host metabolism and identifies novel MRSA targets and suitable vaccine candidates.

#### 2.1.2 Retrieval of non-homologous and essential proteins

Proteins associated with both species metabolic pathways were pooled together, and the proteins that were present in the common pathways were removed, as they interfere with host metabolic pathways. Then the proteins were scrutinized, and only non-homologous MRSA proteins were retained. To achieve this, NCBI BLASTp (Basic Local Alignment Search Tool for protein sequences) tool was utilized against *Homo sapiens* to identify non-host similar proteins, while applying an expected value (E-value) of 0.001, a maximum target sequence of 5,000, and the BLOSUM62 scoring matrix function (https://blast.ncbi.nlm.nih.gov/Blast.cgi?PAGE=Proteins, last accessed July 2025). Afterwards, only the non-homologous proteins were selected for further analysis (Altschul et al., 1997).

Furthermore, non-homologous proteins were queried into the Database of Essential Genes (DEG) to screen essential proteins (http://origin.tubic.org/deg/public/index.php/genome/bacteria, last accessed August 2025). It’s a publicly available repository containing information on essential genes for various organisms. The identification of essentiality is crucial for determining the proteins that are responsible for either MRSA survival or the MRSA mechanism. The built-in analysis tools of DEG are utilized for screening essential proteins, which is useful for discovering effective vaccine candidates. The pathogenic proteins were screened through setting the E-value (10^–5^) and a minimum bit score cut-off of 100, along with the default parameters for sequence identity and coverage as provided by the DEG server. The selection of criteria utilized for the identification of proteins that essential for MRSA survival and pathogenicity, thus prioritizing them as potential vaccine candidates (Zhang et al., 2004).

#### 2.1.3 Prediction of subcellular localization

To understand the functions of proteins for the cellular developmental process and to development of vaccines for particular diseases, it’s essential to access their cellular locations. To address that, the protein’s subcellular localizations were predicted through publicly available web interface tools such as the PSORTb (Protein Subcellular Localization Program for Bacteria) version 3.0 (https://www.psort.org/psortb/, last accessed August 2025). It classified the proteins according to their cellular localizations, including the cell wall, cytoplasmic membrane, cytoplasm, inner membrane, periplasmic space, and outer membrane (Yu et al., 2010). This study focuses on cytoplasmic proteins rather than surface-exposed proteins, which have been primarily targeted in antibody-based vaccines. By prioritizing cytoplasmic proteins, the study aims to elicit T-cell-mediated immunity through endogenous antigen processing and major histocompatibility complex (MHC) class I/II presentation, thereby activating CD8^+^ cytotoxic and CD4^+^ helper T cells (Naorem et al., 2022; Valathoor et al., 2025). Thus, the proteins localized in the cytoplasm of MRSA were utilized for further analysis.

#### 2.1.4 Retrieval of virulence proteins

The proteins were further scrutinized for virulence factor analysis after identifying cytoplasm proteins. For the virulent protein analysis, the virulence factor database (VFDB) (https://www.mgc.ac.cn/VFs/, last accessed August 2025) and virulence factors, information molecules, cellular process, and metabolism prediction (VICMpred) tool were utilized (https://webs.iiitd.edu.in/raghava/vicmpred/, last accessed, August 2025). The VFDB is a free online resource that curates’ bacterial virulence factors and provides experimentally determined information on virulence factor functions, structures, and pathogenic mechanisms, while VICMpred is a web interface that utilizes an support vector machine (SVM)-based algorithm to classify the bacterial proteins into various categories, including virulence factors and molecule information, such as cellular processes and metabolism (Chen et al., 2005; Saha and Raghava, 2006). These web resources potentially identified virulent MRSA protein targets, which could serve as potential candidates for vaccine development.

#### 2.1.5 Homology modelling and protein-protein interactions

Following the retrieval of novel MRSA proteins screened through the metabolic genomic pathway, it's essential to assess the three-dimensional structures to evaluates their biological relevance and functional roles in the context of host-pathogen interactions. If the selected proteins’ 3D structures were not available in the RCSB-PDB (Research Collaboratory for Structural Bioinformatics-Protein Data Bank) (https://www.rcsb.org/), the proteins were modeled using the homology modeling method. For that, SWISS-MODEL (https://swissmodel.expasy.org/, last accessed September 2025), a web server, was utilized for generating 3D structures of the proteins, which aligned the target’s amino acid sequence of proteins with already known experimental structures and estimated the quality of the protein using coverage, E-value, sequence identity, and GMQE (Global Model Quality Estimate) (Waterhouse et al., 2018). After protein structure prediction, the structures’ 3D qualities were validated using various web servers and tools. The tools include PROCHECK, ERRAT, ProSA, and ProQ (https://saves.mbi.ucla.edu/, last accessed September 2025) (Colovos and Yeates, 1993; Wallner and Elofsson, 2003). They are comprehensive computational tools that evaluate the stereochemistry of proteins 3D structures by plotting Ramachandran plots, identifying regions of errors, and providing accuracy rates.

To understand the disease mechanisms, antigen prioritization, and preventive strategies of particular disease-causing proteins, it's crucial to establish their interactions with other molecules, which in turn provide biological insights and molecular mechanisms with nearby proteins or molecules. STRING (Search Tool for the Retrieval of Interacting Genes/Proteins) v11.0 database was utilized for mapping the interactions between non-homologous target proteins to create a protein–protein interaction (PPI) network (https://string-db.org/, last accessed September 2025). It’s a biological database that predicts PPI for known proteins by integrating data from literature resources, genomic data, experimental data, and co-expression data. This analysis provides information about their potential roles in metabolic, biological, and functional aspects (Szklarczyk et al., 2023).

From this phase of analysis, non-homologous, essential, virulent, and novel MRSA proteins were screened, which were further utilized for developing candidate vaccines in phase II analysis.

### 2.2 Multi-epitope vaccine (MEV) construction

#### 2.2.1 Analysis of immunological properties

Initially, immunological properties, focusing on antigenic, allergenic, and toxicity properties, were analyzed for proteins screened from the phase I study. The antigenicity property was assessed through the VaxiJen 2.0 web server (https://www.ddg-pharmfac.net/vaxijen/VaxiJen/VaxiJen.html, last accessed September 2025). It predicts the immunogenic properties of vaccine candidates upon querying their protein sequence (Doytchinova and Flower, 2007). The allergenicity property was analyzed through the AllerTop v2.1 web server (https://www.ddg-pharmfac.net/allertop_test/, last accessed September 2025), which utilized an alignment-free approach and predicts whether the proteins belong to an allergen group that causes any allergic reactions (Dimitrov et al., 2014). On the other hand, ToxinPred used experimental data and machine learning algorithms to classify the toxicity of the protein (http://crdd.osdd.net/raghava/toxinpred/, last accessed September 2025) (S. Gupta et al., 2013).

#### 2.2.2 Predictions of linear B cell and T cell epitopes

To construct targeted vaccines, it’s essential to identify epitopes, which enables the effective, specific, and faster vaccine developments. It determines the precise molecule targets, which stimulates immune responses and minimizes the need for conventional methods. Non-allergen, non-toxic, and antigen-specific linear B-cell and T-cell epitopes were screened through various web interfaces. Identifying B cell epitopes stimulates antibody production, which is important for antibody-based immunity responses, while identifying T cell epitopes recognizes MHC molecules and leads to cell-mediated immunity responses (Shahab et al., 2023). The Immune Epitope Database (IEDB) (https://www.iedb.org/, last accessed September 2025), a user-friendly and freely accessible resource, was utilized for predicting B-cell epitopes; specifically, the tool Bepipred Linear Epitope Prediction 2.0 was employed in the IEDB, which used a random forest algorithm that was trained on identifying linear B-cell epitopes (http://tools.iedb.org/bcell/, last accessed September 2025). On the other hand, the prediction of T-cell epitopes aimed to identify CD4^+^ helper T lymphocytes and CD8^+^ cytotoxic T lymphocytes. Predictions for CD8+/MHC class I/cytotoxic T lymphocytes were conducted using the NetCTLpan 1.1 server (https://nextgen-tools.iedb.org/pipeline?tool=tc1, last accessed September 2025), while the NetMHCIIpan 4.0 server was used for identifying epitopes associated with HLA-II (Human leukocyte antigen) alleles for CD4+/MHC class II/helper T lymphocytes (https://nextgen-tools.iedb.org/pipeline?tool=tc2, last accessed September 2025) (Vita et al., 2025).

#### 2.2.3 Criteria for epitope selection

For the construction of MEV, B cell and T cell epitopes were selected based on the following criteria. Linear B cell epitopes were selected based on their minimum length of greater than or equal to 5 amino acids, as selection of shorter sequences leads to an unstable humoral immune response. In contrast, MHC class I and class II epitopes were ranked according to their predicted binding affinity (IC_50_) values, where the lowest IC_50_ values indicate stronger binding capacity for MHC class I and class II molecules. Specifically, the peptides that bind to MHC class I molecules were predicted using the NetMHCpan 4.1 EL method. The peptide containing IC_50_ ≤ 500 nM and a percentile rank ≤0.5 was considered a strong binder, while the percentile rank above the threshold value was considered a weak binder. In contrast, the peptides that bind to MHC class II molecules were predicted using the recommended NetMHCIIpan 4.1 EL predictor. In this screening, the peptides with IC_50_ ≤ 500 nM and percentile rank ≤2.0 were considered as strong binders, which were subsequently utilized for further vaccine constructions (Sethi et al., 2024). Following the retrieval of B and T cell epitopes, they were further scrutinized for analyzing antigenicity, allergenicity, and toxicity properties through VaxiJen, AllerTop, and ToxinPred tools. The epitopes predicted as antigen, non-allergen, and non-toxic were utilized for further interferon analysis. The interferon gamma (INFγ) producing potential of the selected T cell epitopes was predicted using the IFNepitope web server (https://webs.iiitd.edu.in/raghava/ifnepitope/index.php, last accessed December 2025). This analysis was used to evaluate the ability of the selected T helper cell epitopes to elicit T helper cell-mediated cellular immune responses, which are essential for effective protection against intracellular pathogens. For screening INFγ-producing peptides, default parameters and a support vector machine (SVM)-based approach were employed. Through this algorithm, the web server classifies peptides as INFγ-inducers (positive score >0) and INFγ-non-inducers (negative score <0).

#### 2.2.4 Prediction of population coverage

To construct an effective multi-epitope vaccine, it is crucial to assess the potential ability of alleles worldwide. Since HLA alleles (Class I and Class II) are classified as highly polymorphic, they are differently distributed among various ethnic groups. In this study, the population coverage was evaluated worldwide and in India using selected B cell and T cell epitopes. The IEDB population coverage web interface was used for determining the fraction of the worldwide and Indian population coverage upon querying selected epitopes (http://tools.iedb.org/population/, last accessed September 2025). These analyses ensure the applicability and validation of the selected epitopes in multiple groups of populations (Garrido-Palazuelos et al., 2024).

#### 2.2.5 MEV construction and physicochemical characterization

The selected B cell and T cell epitopes, which have amino acid sequences with high population coverage, were used for designing a multi-epitope vaccine along with adjuvants and linkers. A serial arrangement of the vaccine was designed with 50S ribosomal subunit protein as an adjuvant, which enhances immune response by activating the innate immune system. Following that, every B and T cell epitope was linked with the help of flexible linkers such as EAAAK, GPGPG, and AYY (Sethi et al., 2024). These linkers will improve the protein stability and immunogenicity. To validate the physicochemical properties of the designed vaccine, the Expasy ProtParam server was used (https://web.expasy.org/protparam/, last accessed September 2025). It uses known physical and chemical properties of individual amino acids, which are stored in the UniProt database, and provides the output of molecular weight, estimated half-life, isoelectric point, atomic composition, instability index, and so on (Walker, 2005). Further, the constructed vaccine’s antigenicity, allergenicity, and toxicity were evaluated with VaxiJen, AllerTop, and ToxinPred web servers.

#### 2.2.6 Prediction of secondary and tertiary structure

The designed vaccine’s secondary structure was predicted using the PSIPRED web server, which predicts alpha helices, beta sheets, and coils of the queried amino acid sequences by using feed-forward neural networks and the position-specific iterated BLAST algorithm (https://bioinf.cs.ucl.ac.uk/psipred/, last accessed September 2025) (McGuffin et al., 2000). The tertiary structure of the constructed vaccine was predicted through the trROSETTA web server, which generates the 3D structure based on deep learning methods (https://yanglab.qd.sdu.edu.cn/trRosetta/, last accessed September 2025) (Du et al., 2021). Then, the modeled structure’s quality was validated using comprehensive computational tools such as PROCHECK, ERRAT, ProSA, and ProQ, which evaluate the 3D structure by visualizing the Ramachandran plot and generating accuracy and error rates.

#### 2.2.7 Molecular docking of MEV vaccine against TLR receptors

To determine the molecular interactions with immune receptors, the constructed vaccine was docked against toll-like receptors, including TLR2 (PDB ID: 6NIG) and TLR 4 (PDB ID: 2Z63). These immune receptors are selected based on their vital role in recognizing pathogen-associated molecular patterns and to activate immune responses, especially in bacterial components such as lipoproteins (TLR2) and lipopolysaccharides (TLR4). Nevertheless, they also initiate downstream signaling cascades, which could activate innate immune systems and lead to the production of proinflammatory cytokines and interleukins, thereby interconnecting innate and adaptive immunity responses (Kumar et al., 2024). For this docking analysis, the ClusPro server 2.0 was used with default parameters, which provides the binding affinity between the vaccine and immune receptors (https://cluspro.bu.edu/login.php?redir=/home.php, last accessed September 2025). Subsequently, protein-protein interaction was examined through the PDBsum server (https://www.ebi.ac.uk/thornton-srv/databases/pdbsum/, last accessed September 2025). This comprehensive analysis provides information on hydrogen bonds, non-bonded interactions, and salt bridge interactions (Kozakov et al., 2017; Laskowski et al., 1997). From the docking results, the toll-like receptor that had a highest binding affinity, high number of hydrogens, and salt bridge interactions with the constructed vaccine was utilized for further analysis.

#### 2.2.8 Molecular dynamic simulation of the vaccine-receptor complex

Molecular dynamic simulation (MDS) was carried out using GROMACS v2023.5 with the CHARMM27 atom force field to further confirm the structural stability of the designed vaccine with immune receptors (https://www.gromacs.org/). The complex and apo-form of the vaccine were solvated in the cubic simulation box, maintaining a minimum distance of 1.0 nm between the protein surface and the box edges, and solvated with the simple point charge (TIP3P) water model. The system was neutralized using chloride or sodium ions and energy minimization was carried out using the steepest descent algorithm, where the minimization step was paused at 10.0 kJ/mol. Subsequently, the system underwent equilibration under the conditions of a fixed number of particles, volume, and temperature (NVT) as well as a fixed number of particles, pressure, and temperature (NPT) for a duration of around 100 picoseconds, where the pressure of the system was maintained using the Parrinello-Rahman technique, while the temperature was kept at 300 K using the Berendsen thermostat method for every 2 femtoseconds. After neutralizing the system, the vaccine-TLR complex was subjected to a 100 ns simulation (Lemkul, 2019; Munieswaran et al., 2025). Following that, MDS parameters such as root mean square deviation (RMSD), radius of gyration (ROG), and solvent accessible surface area (SASA) were calculated and visualized using Xmgrace software for both the apo-form vaccine and the complex.

#### 2.2.9 Codon adaptation and *in silico* cloning

To optimize vaccine gene expression in the chosen host and to validate the vector insertion for the feasibility of laboratory cloning, codon optimization and *in silico* cloning were performed. Reverse translation and codon optimization were determined through the Java Codon Adaptation Tool (JCAT), which adapts the codon usage for a gene to a specific host organism by optimizing synonymous codon usage (https://www.jcat.de/, last accessed September 2025). Through this server, CAI (codon adaptive index) and vaccine GC content percentage were calculated, which in turn improves the heterologous protein production (Grote et al., 2005). After optimization, the sequence was cloned into the *E. coli* vector (pET28a(+)) using the SnapGene software (https://www.snapgene.com/, last accessed September 2025). It’s used in molecular biology to designs and simulates numerous *in silico* cloning techniques, such as TOPO (topoisomerase), Gateway, Gibson assembly, and TA/GC cloning, and visualizes them (Samad et al., 2020).

#### 2.2.10 Immune profiling of MEV vaccine

The immune response of the constructed vaccine was evaluated through the C-Immsim web server, which simulates the natural immune response in the human body by employing PSSM (position-specific scoring matrices) and machine learning techniques (https://kraken.iac.rm.cnr.it/C-IMMSIM/index.php?page=1, last accessed September 2025). Immune simulation for the constructed vaccine was performed with three injections at time intervals of 0, 28, and 56 days, where each injection contains 1,000 vaccine proteins without lipopolysaccharide (LPS), which mimic multiple vaccine doses. The simulation steps parameter was set to be 1,050, which corresponds to approximately 365 days, while the remaining parameters, such as random seed, simulation volume, and host HLA selection, were kept at default parameters (Rapin et al., 2010).

## 3 Results

### 3.1 Phase I

The primary objective of phase I is to identify novel immunogenic targets of MRSA through subtractive metabolic pathway analysis. The protein was selected based on the following criteria: being non-homologous to host proteins, representing a novel target, being an essential protein for bacterial mechanisms, exhibiting virulence properties, and playing a significant role in the overall metabolic pathway of MRSA infection.

#### 3.1.1 Identification of unique and non-homologous proteins

Initially, the metabolic pathways of *Homo sapiens* and MRSA were retrieved from the KEGG database. A total of 357 metabolic pathways for *H. sapiens* and 109 MRSA metabolic pathways were retrieved. The whole proteome of *H. sapiens* and MRSA are given in Supplementary Material Table 1. Among them, 27 unique metabolic pathways of MRSA were manually identified by comparing both metabolic pathways. Table 1 presents the unique metabolic pathways of MRSA. Through analyzing the unique metabolic pathways, a total of 294 proteins were identified, and they were subsequently screened for identifying non-homologous proteins. The unique metabolic pathway proteins are given in Supplementary Material Table 2. Each protein from unique metabolic pathways was compared with *H. sapiens*. By analyzing the results, a total of 180 proteins were categorized as homologous to the host, while 114 proteins were categorized as non-homologous. These non-homologous proteins were utilized for further screening processes.

**TABLE 1:** Unique metabolic pathways of MRSA.

S. No	KEGG ID	Unique metabolic pathways
1	sau00261	Monobactam biosynthesis
2	sau00300	Lysine biosynthesis
3	sau00362	Benzoate degradation
4	sau00401	Novobiocin biosynthesis
5	sau00460	Cyanoamino acid metabolism
6	sau00521	Streptomycin biosynthesis
7	sau00541	O-Antigen nucleotide sugar biosynthesis
8	sau00542	O-Antigen repeat unit biosynthesis
9	sau00543	Exopolysaccharide biosynthesis
10	sau00550	Peptidoglycan biosynthesis
11	sau00552	Teichoic acid biosynthesis
12	sau00622	Xylene degradation
13	sau00625	Chloroalkane and chloroalkene degradation
14	sau00626	Naphthalene degradation
15	sau00643	Styrene degradation
16	sau00660	C5-Branched dibasic acid metabolism
17	sau00680	Methane metabolism
18	sau00906	Carotenoid biosynthesis
19	sau00907	Pinene, camphor and geraniol degradation
20	sau00997	Biosynthesis of various other secondary metabolites
21	sau00998	Biosynthesis of various antibiotics
22	sau00999	Biosynthesis of various plant secondary metabolites
23	sau02020	Two-component system
24	sau02024	Quorum sensing
25	sau02040	Flagellar assembly
26	sau02060	Phosphotransferase system (PTS)
27	sau03070	Bacterial secretion system

#### 3.1.2 Identification of essential proteins and subcellular loculations

**FIGURE 2:**
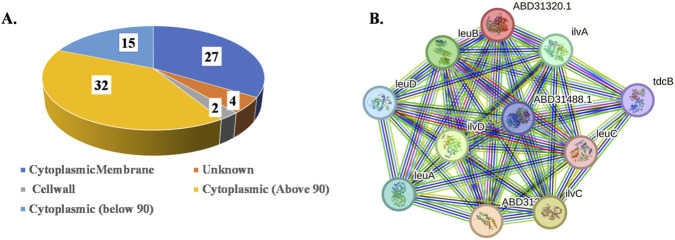
Subcellular localization and protein-protein interaction, **(A)** a pie chart represents the distribution of subcellular localization of shortlisted essential, non-homologous, pathogen-specific proteins predicted through PSORTb. **(B)** Protein-protein interaction analysis for selected ALS protein. The STRING results showed different interactions of proteins with each other represented by nodes and edges.

To evaluate the screened proteins essentiality in MRSA, the DEG database was utilized with default parameters. From this analysis, 81 proteins were identified as essential proteins that were necessary for MRSA survival and pathogenesis. Following that, the subcellular localization of the screened proteins revealed that 27 proteins were located in the cytoplasmic membrane, 2 proteins were present in the cell wall, 4 proteins were predicted in the unknown location, and the remaining 47 proteins were found in the cytoplasm. Figure 2A illustrates the localization of the screened proteins. This prediction showcased that the majority of the proteins were located in the cytoplasm. Among them, the proteins that scored above 90% were categorized as highly reliable. By analyzing the prediction scores, 32 proteins passed the criteria, which qualified them for further screening. In contrast, 15 proteins had scores below 90%, which were excluded from the following analysis.

#### 3.1.3 Identification of virulence proteins

After determining subcellular localizations, 32 proteins were subjected to virulent factor analysis. The results revealed that only one protein passed all the criteria, namely, acetolactate synthase (ALS), a protein that plays a vital role in the MRSA metabolism and survival. Upon analyzing all the parameters, this ALS protein was declared as a novel, non-homologous, essential, and virulent target in the MRSA organism. Table 2 shows a list of screened non-homologous, essential, and virulent proteins.

**TABLE 2:** Non-homologous, essential, and virulent targets of MRSA.

S. No	UniProt id	Protein name	PSORTp	Score	AA length	Gene	VFDB	VICMpred
1	Q7A5H6	Transcriptional regulatory protein SrrA	Cytoplasmic	10	241	srrA	No	Metabolism
2	P60610	Transcriptional regulatory protein LytR	Cytoplasmic	10	246	lytR	No	Cellular process
3	P99143	Phosphocarrier protein HPr	Cytoplasmic	10	88	ptsH	No	Metabolism
4	A0A0H3JND9	KDP operon transcriptional regulatory protein KdpE	Cytoplasmic	9.97	231	kdpE	Yes	Cellular process
5	Q7A7X9	Transcriptional regulatory protein HptR	Cytoplasmic	9.97	252	hptR	Yes	Cellular process
6	Q99V14	Phosphoenolpyruvate-protein phosphotransferase	Cytoplasmic	9.97	572	ptsI	No	Metabolism
7	Q7A5P7	2,3,4,5-tetrahydropyridine-2,6-dicarboxylate N-acetyltransferase	Cytoplasmic	9.97	239	dapH (dabD)	Yes	Cellular process
8	A0A0H3JMW3	UDP-N-acetylmuramoyl-tripeptide--D-alanyl-D-alanine ligase	Cytoplasmic	9.97	452	murF	No	Virulence factors
9	Q7A7B4	Bifunctional protein GlmU	Cytoplasmic	9.97	450	glmU	Yes	Cellular process
10	A0A0H3JKC9	Capsular polysaccharide synthesis enzyme Cap5G	Cytoplasmic	9.97	374	capG	No	Metabolism
11	P67765	Serine acetyltransferase	Cytoplasmic	9.97	213	cysE	No	Metabolism
12	P0A090	UDP-N-acetylmuramoylalanine--D-glutamate ligase	Cytoplasmic	9.97	449	murD	Yes	Cellular process
13	P63892	D-alanine--D-alanine ligase	Cytoplasmic	9.97	356	ddl	Yes	Cellular process
14	P65480	UDP-N-acetylmuramoyl-L-alanyl-D-glutamate--L-lysine ligase	Cytoplasmic	9.97	494	murE	Yes	Metabolism
15	A0A0H3JV81	Acetolactate synthase	Cytoplasmic	9.97	589	ilvB	Yes	Virulence factors
16	Q99TF2	Acetate kinase	Cytoplasmic	9.97	400	ackA	Yes	Metabolism
17	P64270	2,3-Bisphosphoglycerate-independent phosphoglycerate mutase	Cytoplasmic	9.97	505	gpmI	Yes	Cellular process
18	A0A0H3JUI4	Alkaline phosphatase synthesis transcriptional regulatory protein	Cytoplasmic	9.97	234	phoP	No	Cellular process
19	A0A0H3JKS9	KdpE(SCCmec) protein	Cytoplasmic	9.97	231	kdpE(SCCmec)	No	Cellular process
20	Q7A8E1	Transcriptional regulatory protein WalR	Cytoplasmic	9.97	233	walR	No	Cellular process
21	Q99VW2	Response regulator protein GraR	Cytoplasmic	9.97	224	graR	No	Cellular process
22	Q99U73	Response regulator ArlR	Cytoplasmic	9.97	214	arlR	No	Cellular process
23	A0A0H3JLT1	SA1159 protein	Cytoplasmic	9.97	200	-	Yes	Cellular process
24	Q7A4R9	Response regulator protein VraR	Cytoplasmic	9.97	209	vraR	No	Cellular process
25	Q7A3U5	Oxygen regulatory protein NreC	Cytoplasmic	9.97	217	nreC	No	Metabolism
26	A0A0H3JKX9	Anthranilate synthase component 1	Cytoplasmic	9.97	468	-	No	Cellular process
27	A0A0H3JKS9	KdpE(SCCmec) protein	Cytoplasmic	9.97	231	kdpE(SCCmec)	Yes	Cellular process
28	Q99TT5	RNA polymerase sigma factor SigA	Cytoplasmic	9.97	368	sigA	Yes	Information and storage
29	A0A0H3JMK4	Aspartate-semialdehyde dehydrogenase	Cytoplasmic	9.67	329	asd	Yes	Metabolism
30	P63894	4-Hydroxy-tetrahydrodipicolinate reductase	Cytoplasmic	9.67	240	dapB	Yes	Cellular process
31	A0A0H3JN19	Riboflavin biosynthesis protein RibD	Cytoplasmic	9.67	347	ribD	Yes	Cellular process
32	A0A0H3JS31	SA2418 protein	Cytoplasmic	9.67	221	-	No	Cellular process

#### 3.1.4 Acetolactate synthase

Through subtractive genomics, acetolactate synthase (ALS) was identified as a novel, potentially virulent, and therapeutic target in MRSA. ALS plays a major role in catalyzing the first metabolic step of the branched-chain amino acid biosynthesis pathway by condensing approximately 2 pyruvate molecules into 2-acetolactate along with CO_2_ release (Q. Zhou et al., 2007). This reaction is predominately dependent on the cofactor, namely, thiamine pyrophosphate (TPP), and generates reactive intermediates upon deprotonation. These intermediates further targets carbonyl carbon of pyruvate, facilitating decarboxylation, and adequately forms a covalent complex. Following that, complex interacts with second pyruvate molecule to yield acetolactate while regenerating TPP. Thus, targeting ALS protein in MRSA may perturb branched-chain amino acid biosynthesis by affecting the growth, virulence, and resistance mechanisms of MRSA. Additionally, targeting the TPP-dependent catalytic mechanism in ALS represents a promising strategy for the development of novel antimicrobials (Naidu, 2023).

#### 3.1.5 Homology modelling and protein-protein networks

**FIGURE 3:**
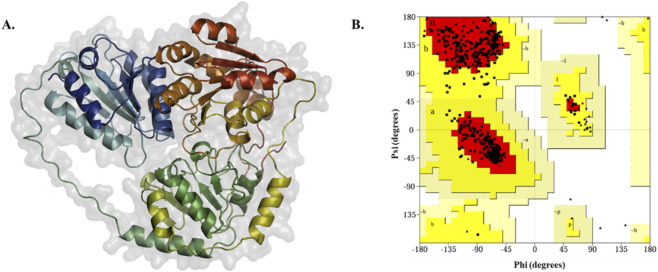
Modeled 3D structure of ALS protein and structure evaluation. **(A)** modeled 3D structure of ALS protein. **(B)** 3D structure validation through Ramachandran plot using ProCheck webserver.

The homology model was constructed for ALS proteins, as selected protein’s 3D structure was not available in the RCSB-PDB database. Primarily, ALS protein’s FASTA sequence was retrieved from the UniProt database with ID of A0A7U7EW44, containing 589 amino acids. It has 3 domains, including thiamine pyrophosphate enzyme N-terminal TPP-binding (36–151 aa), thiamine pyrophosphate enzyme central (224–359 aa), and thiamine pyrophosphate enzyme TPP-binding (417–565 aa). The sequence was queried through SWISS-MODEL, and the top-scored protein’s 3D structure was utilized for structure validation and quality assessment. The modeled protein was validated through plotting a Ramachandran plot, where most of the residues were present in the favoured regions with a value of 91.3%, suggesting good and well-stereochemical quality. On the other hand, ERRAT showed a 92.47% overall quality factor, indicating satisfactory non-bonded atomic interactions, whereas ProSA and ProQ analysis exhibited a high-quality constructed ALS protein. The Figures 3A,B illustrates the 3D structure and Ramachandran plots of ALS protein, and the Table 3 presents the validation results for the modeled protein structure.

**TABLE 3:** Structure validation of modelled ALS protein.

Structure validation			
ProCheck (Ramachandran plot)	Residues in most favoured regions [A,B,L]	538	91.3%
	Residues in additional allowed regions [a,b,l,p]	50	8.7%
	Residues in generously allowed regions [∼a,∼b,∼l,∼p]	0	0.0%
	Residues in disallowed regions	0	0.0%
ERRAT	Overall model quality (non-bonded atomic interaction)	92.4731	
ProSA (Z score)	Over all model quality (energy overall energy profile)	−11.2	
ProQ (protein quality predictor)	LG-score	9.259	
	MaxSub	0.5	

Analysis of PPI network revealed that the ALS protein (represented by ABD31320.1) interacts with several key enzymes, which predominately play a role in the branched-chain amino acid (BCAA) biosynthesis pathway. The proteins, including ilvC, ilvD, leuA, leuB, leuC, leuD, and tdcB, were provided the average node degree of ∼9.45 and demonstrated more edges (52) than the expected node degree and edges (12), suggesting a strong functional correlation with the selected ALS protein. Further, gene ontology enrichment analysis highlighted those interconnected proteins consistently contributed to the synthesis of BCAA (isoleucine, valine, and leucine) and exhibited enzymatic functions such as acetolactate synthase activity, dehydratase activity, lyase activity, magnesium ion binding, and vitamin binding, which were located in the cytosol of MRSA. The literature resources revealed that these PPIs link to MRSA bacterial growth, virulence, and various metabolism pathways, such as 2-oxocarboxylic acid metabolism and C5-branched dibasic acid metabolism. This evidence suggested that this PPI integration plays a major role in central carbon metabolism. Thus, analyzing the PPI networks revealed that ALS acted as a metabolic hub protein that was essential for MRSA survival, which could be exploitable for the development of preventive measures using reverse vaccinology. Figure 2B show the protein -protein interaction network of ALS using STRING database.

### 3.2 Phase II

#### 3.2.1 Selection and screening of B cell and T cell epitopes

Initially, the selected ALS protein from the phase I study was scrutinized for antigenicity, allergenicity, and toxicity using VaxiJen, AllerTop, and ToxinPred web servers. From this analysis, the selected ALS protein was considered as a portable antigen (0.61), a non-allergen, and a non-toxin, suggesting that ALS is suitable for constructing a multi-epitope vaccine.

For the construction of MEV, B cell and T cell epitopes should be selected, which are responsible for activating innate and adaptive immunity responses. Through the Bepipred Linear Epitope Prediction 2.0 server, around 9 linear B-cell epitopes with scores ranging from 0.5 to 1.0 were predicted (Supplementary Material Table 3). Out of 9, only 3 of the epitopes were categorized as non-toxic, antigenic, and non-allergenic. These epitopes were considered for further analysis. Following that, a total of 183 CTL (cytotoxic T lymphocytes) epitopes and 473 HTL (helper T lymphocytes) epitopes were predicted through NetCTL 1.2 and NetMHC II 2.3 servers. The overall epitopes of MHC class I and II are given in Supplementary Material Table 4, 5. Among these epitopes, only 11 from CTL and 36 from HTL were considered non-toxic, antigenic, and non-allergenic. From these, 8 high-affinity HTL epitopes restricted to MHC class II were selected for final construction of MEV, as they exhibited low IC_50_ values (best binding affinity) and favourable immunogenic properties. Further, the analysis of the INFγ-producing T-cell epitopes revealed that among the 19 selected T-cell epitopes, 17 were predicted as INFγ inducers, which scored above the threshold value of 0, while the remaining two epitopes were predicted as non-INFγ inducers based on the negative SVM scores (Supplementary Material Table 6). Precisely, the epitopes that bind to MHC class II molecules were scored, ranging from 0.0017 to 0.6531, demonstrating their capacity to elicit the T-helper (Th1) cell-mediated cellular immunity. These results suggested that the selected T-cell epitopes were suitable for constructing MEV.

#### 3.2.2 Population coverage

**FIGURE 4:**
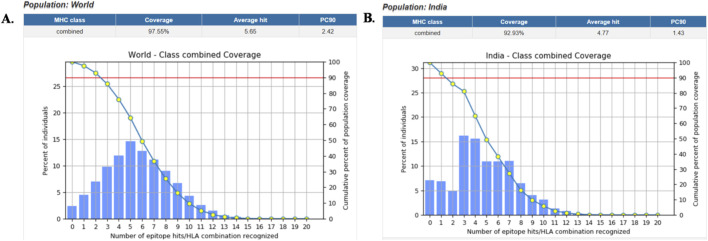
Population coverage analysis of the designed ALS vaccine, **(A)** worldwide and **(B)** India population coverage.

To estimate the population coverage of the designed MEV, IEDB tools were utilized, which assessed both MHC class I and II restricted alleles at the global level and within the Indian population. It was found that 97.55% of the global population and 92.93% of the Indian population were covered by selected T cell epitopes. The Figures 4A,B represents the global and Indian population coverage. Further, the selected T-cell epitopes provide a broad class I and II coverage. The predicted population coverage revealed that CTL epitopes bind to prevalent HLA-A such as HLA-A*02:01, HLA-A*02:03, HLA-A*02:06, HLA-A*11:01, HLA-A*24:02, and HLA-A*68:01 and additionally contributes with HLA-B such as HLA-B*44:02/03, HLA-B*15:01, HLA-B*35:01, and HLA-B*53:01 alleles. In contrast, HTL epitopes provided strong binding with multiple alleles, including DRB1*03:01, DRB1*04:01/04, DRB1*07:01, DRB1*09:01, DRB1*10:01, and DRB1*15:01. These results suggested that the high population coverage of predicted CTL and HTL epitopes had strong potential ability to be widely recognized by diverse HLA alleles, suggesting their effectiveness in the development of a vaccine against the targeted pathogen.

#### 3.2.3 MEV construction

**FIGURE 5:**
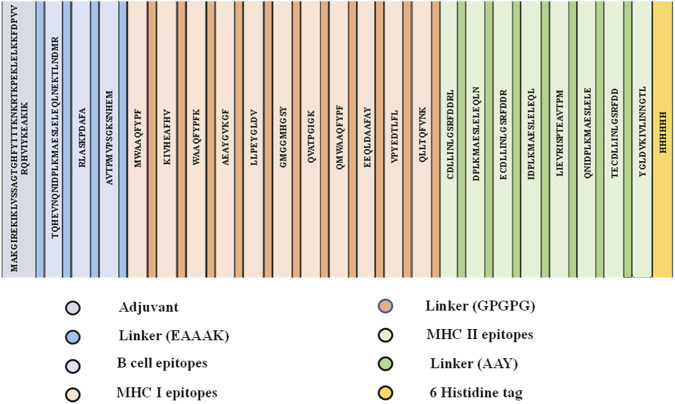
Primary structure of constructed ALS vaccine.

The final ALS vaccine was constructed with a suitable adjuvant and appropriate linkers. Initially, 50S ribosomal subunit protein with a UniProt ID of P0A7N9 was added as an adjuvant, which has been employed by researchers to induce dendritic cell maturation and increase the production of pro-inflammatory cytokines (TNF-α and IL 6), thereby increasing immunomodulatory activity when linked to toll-like receptors. Concurrently, B cell epitopes and CTL and HTL epitope linkers such as EAAAK, GPGPG, and AAY were included to reduce unfavorable interactions, improve stability, and facilitate proteasomal cleavage, thereby maintaining conformational dynamics between epitopes (P. Zhou et al., 2025). Among the linkers, EAAAK was a rigid linker, capable of forming an alpha helix at the amine terminal, which linked B cell epitopes and the adjuvant. Subsequently, the GPGPG linker was employed to connect the CTL epitopes and B cell epitopes. On the other hand, the AAY linker was used for interconnecting CTL and HTL epitopes. At the end, six histidine tags (6His) were added to facilitate efficient purification and detection of recombinant vaccine protein without disrupting antigenicity properties. The Figure 5 illustrates overall ALS MEV construction using adjuvants and linkers.

Initially, the physicochemical properties of the constructed ALS vaccine were examined using the ProtParam server. From the analysis, the total number of amino acids in the ALS vaccine was determined to be 433, and the observed molecular weight was 46,571.02 kDa. In contrast, the total number of negatively charged residues containing ASP and GLU and positively charged residues (ARG and LYS) were observed to be 52 and 39, respectively. The instability index was predicted to be 31.32, which classified the constructed ALS vaccine as stable. Grand Average of Hydropathicity (GRAVY) was computed to be −0.320, indicating ALS had a strong hydrophilic nature. Further, the analysis of immunological properties revealed that the ALS vaccine was predicted as probable antigen (0.7239), non-allergen, and non-toxin. Table 4 represents the immunological and physicochemical properties of the constructed ALS vaccine.

**TABLE 4:** Immunological and physicochemical properties of constructed ALS vaccine.

S. No	Physicochemical and immunological properties	Value
1	Antigenicity	0.7239 (probable antigen)
2	Allergenicity	Non-allergen
3	Toxicity	Non-toxic
4	Number of amino acids	433
5	Theoretical pI	5.50
6	Molecular weight (KDa)	46,571.02
7	Total number of negatively charged residues (Asp + Glu)	52
8	Total number of positively charged residues (Arg + Lys)	39
9	Formula	C_2101_H_3226_N_552_O_615_S_16_
10	Total number of atoms	6,510
11	Instability index	31.32 (stable)
12	Aliphatic index	75.84
13	Grand average of hydropathicity (GRAVY)	−0.320

#### 3.2.4 Prediction of secondary and tertiary structure

**FIGURE 6:**
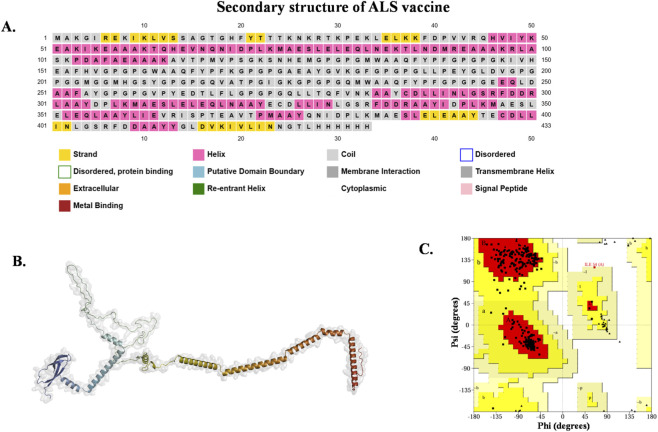
2D and 3D structure constructed vaccine. **(A)** 2D structure of ALS vaccine. **(B)** 3D structures of ALS vaccine and **(C)** the modeled structure validated through Ramachandran plot.

The secondary structure of the ALS vaccine was predicted through the PSIPRED server, which determined its folding nature. Upon reviewing the results (Figure 6A), the majority of the ALS vaccine contains coils (63.4%), and the remaining 2D structures, such as alpha helix and beta strand, had predicted scores of 20% and 15.6%, respectively. Following that, the tertiary structure of the ALS vaccine (Figure 6B) was constructed through the trROSETTA server. Furthermore, the 3D structure was validated using a Ramachandran plot (Figure 6C). The analysis revealed that most of the residues (325) were found in favored regions, which account for 95.3%. The remaining residues were found in allowed regions, accounting for 0.4%. This confirms the good stereochemical quality of the ALS vaccine. Additionally, the overall quality factor was determined to be 96.988, which was calculated using the ERRAT server, suggesting reliable non-bonded interactions. Subsequently, ProSA validation produced a Z-score of −2.56, which falls within the range of experimentally determined protein structures, while ProQ predicted high model reliability with an LG-score of 9.248 and a MaxSub score of 0.502. These results confirmed that the constructed ALS vaccine had adequate quality and was structurally valid, which could be utilized for further studies.

#### 3.2.5 Binding affinity analysis

**FIGURE 7:**
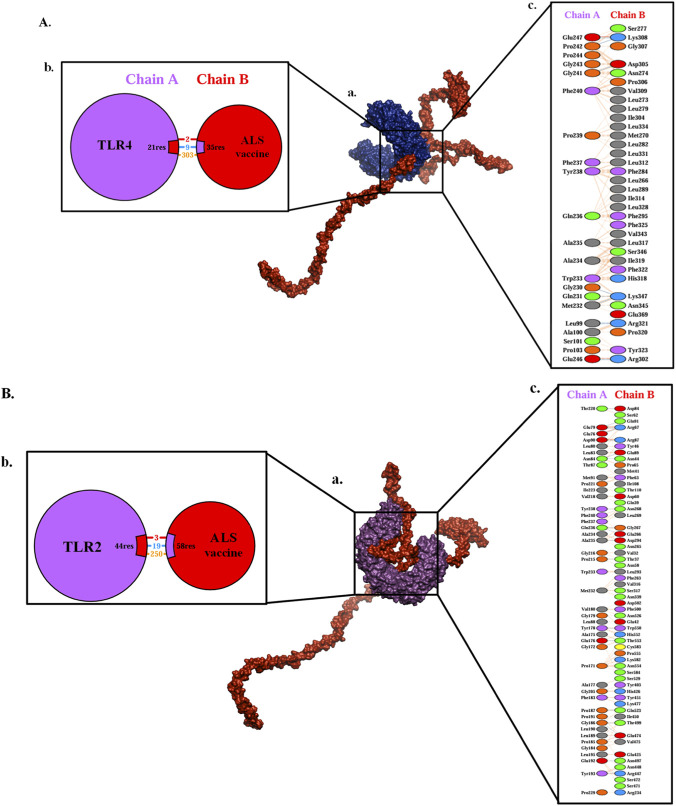
Protein-protein interaction between the constructed vaccine and immune receptors. **(A)**, (a) the complex of the ALS vaccine and TLR2, (b) the total interaction between the vaccine and TLR2 (with colors red, blue, and orange indicating salt bridges, disulphide bonds, and non-bonded contacts, respectively), and (c) residue interactions across the interface. **(B)**, (a) the complex of the ALS vaccine and TLR4, (b) the total interaction between the vaccine and TLR4 (with colors red, blue, and orange indicating salt bridges, disulphide bonds, and non-bonded contacts, respectively) and (c) residue interactions across the interface.

The immune responses of the ALS vaccine against toll-like receptors were performed using molecular docking analysis. The docking results revealed that the ALS had higher binding affinity towards TLR4 (−1,438.7 kcal/mol) (Figure 7A) than TLR2 (−1,103.5 kcal/mol) (Figure 7B), suggesting the potential ability of the ALS vaccine to effectively activates the TLR4 mediated innate immune signaling. Then, the interactions between the docked complexes were visualized using the PDBsum web server. The TLR2 had 2 salt bridges, 9 disulfide bonds, and 303 non-bonded interactions with the ALS vaccine, while, TLR4-ALS exhibited 3 salt bridges, 19 hydrogen bonds, and 250 non-bonded interactions. Based on the more favorable binding affinity score, the TLR4-ALS complex was selected for further analysis.

#### 3.2.6 Molecular dynamic simulations

**FIGURE 8:**
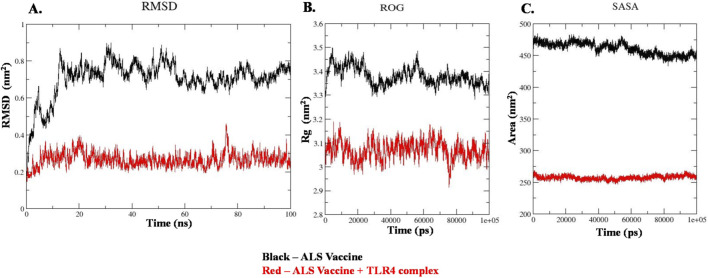
Molecular dynamic simulation analysis of **(A)**, RMSD **(B)**, ROG, and **(C)**, SASA graphs. The graphs represent of ALS vaccine and ALS vaccine + immune receptor (TLR4) complex. Black indicates the apo form of ALS vaccine and red color indicates ALS vaccine and TLR4 complex.

The structural stability of the TLR4-ALS complex was performed through molecular dynamics simulation studies. Calculating MDS parameters such as RMSD, ROG, and SASA offers a greater understanding of structural stability and motions at the atomic and molecular level. The Figure 8A showcases the RMSD of the vaccine complexes. By examining the results, the ALS vaccine (apo form) alone exhibited higher fluctuations in the range of 0.2–0.85 nm. In contrast, the ALS-TLR4 complex exhibited stable regions followed by minimum fluctuations observed within the range of 0.2–0.4 nm. These results suggested that the ALS vaccine-TLR4 complex had a more stable conformation during the 100 ns simulation. Following that, the compactness of the ALS complexes was determined through plotting an ROG graph. The Figure 8B illustrates the ROG of the complex and the ALS vaccine. From the graph, the TLR4-ALS complex consistently displayed lower ROG values (3.3–3.5 nm) when compared to the ALS vaccine (3.0–3.2 nm). This analysis suggested that the TLR4-ALS complex had more compactness and cohesive structure assembly. Concurrently, the solvent accessibility of the complexes was determined through plotting an SASA graph (Figure 8C). The graph revealed that the ALS vaccine-TLR4 complex had less exposure to the solvent (450–475 nm) compared to the apo-form vaccine complex, which was found in the range of 250–265 nm. The decreased SASA value analyzed for the complex indicates that the residues become buried at the interaction interface and further confirms its stability over 100 ns simulations. Overall, the calculated MDS parameters exhibited that the TLR4-ALS vaccine had enhanced stability, compactness, and less solvent exposure over time, which make it suitable for an appropriate vaccine candidate.

#### 3.2.7 Codon adaptation and *in silico* cloning

**FIGURE 9:**
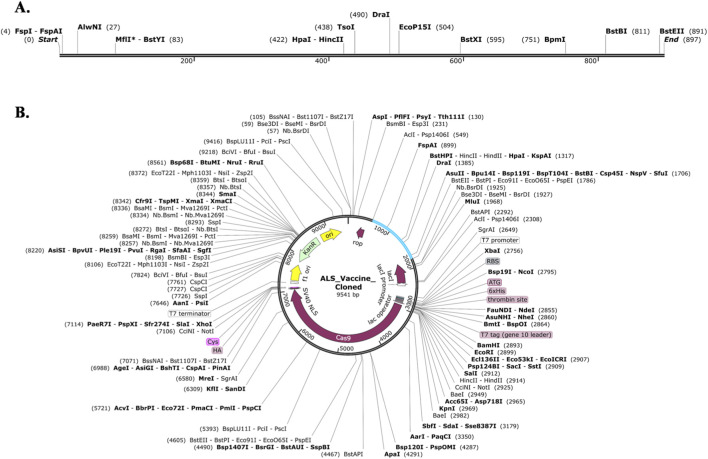
*In silico* cloning of was employed to insert the vaccine design pET28a (+) expression vector. **(A)** Restriction sites of designed vaccine **(B)** cloned vaccine (blue color) in pET28a (+) vector.

To improve the translation efficiency of a target gene, codon optimization was performed to minimize host-specific codon bias. The Java Codon Adaptation tool was utilized to optimize the codon usage of the ALS vaccine construct in *E. coli* (K12 strain) and generates 1,298 bp of DNA sequence. The optimized CAI was found to be 0.98, indicating strong potential for expression, and a GC content was found to be 55.67%, which lies within the ideal range (30%–70%). Further, the optimized DNA sequence was cloned into the pET-28 (+) vector between the BstEII and AclI restriction sites, which were absent in the construct and thus added at the N-terminal and C-terminal ends of the ALS vaccine. Finally, the *in silico* cloning was successfully performed using the SnapGene software, and the total length of the final vaccine clone was observed to be 9,541 bp. The Figures 9A,B illustrates restriction sites of the designed vaccine and cloned ALS vaccine in pET-28 (+) vector.

#### 3.2.8 Immune simulation

**FIGURE 10:**
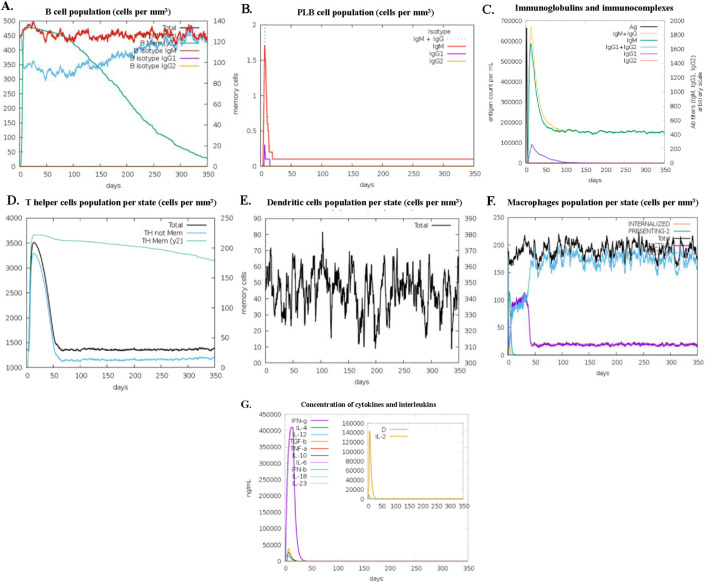
Immune response profile of the designed vaccine. **(A)** B cell population (cells per mm^3^), **(B)** PLB cell population (cells per mm^3^), **(C)** concentration of Immunoglobulins and immunocomplexes, **(D)** T helper cells population per state (cells per mm^3^), **(E)** Dendritic cells population per state (cells per mm^3^), **(F)** Macrophages population per state (cells per mm^3^), **(G)** concentration of cytokines and interleukin.

The immune responses simulated through the C-ImmSim server demonstrated quantitative stability and immune activation for the administered ALS vaccine. Precisely, the total B cell population elevated rapidly and stabilized between 420 and 500 cells/mm^3^, while the memory B cells gradually increased and reached a peak of 130–140 cells/mm^3^ (Figure 10A). This suggested that the proposed ALS vaccine may have the ability to activate sustained humoral immunity. Further, the plasma B cell population (Figure 10B) revealed the transient peak in the range of 1.5–1.7 cells within 15 days and a decline observed afterwards, indicating efficient antibody clearance and controlled antibody secretion. Analysis of immunoglobulin production demonstrated that the secondary and tertiary immune responses exhibited elevated levels of IgM + IgG, IgM, IgG1 + IgG2, and IgG1 antibody productions for 200 days, confirming its long-term antibody-mediated immunity (Figure 10C). T-helper cell population (Figure 10D) peaked in the range of 1,300–3,800 cells/mm^3^ and became stabilized at around 1,300 to 1,500 cells/mm^3^. Dendritic cells (Figure 10E) exhibited minimal fluctuations across simulations in the range of 20–80 cells/mm^3^, while macrophages (Figure 10F) exhibited sustained antigen internalization and MHC class II presentation by maintaining presenting cell populations in the range of 150–200 cells/mm^3^. Furthermore, the concentration of the antigen was significantly decreased after 40 days, while an antibody concentration was progressively increased during each phase of the immune response. In addition to enhanced antibody production, elevated levels of IFN-γ were also observed during immunological reactions (Figure 10G). Overall, these immune response results confirmed that the designed ALS vaccine had the potential to trigger both a robust cellular and humoral immune response in the host organism.

## 4 Discussion

Despite the limited availability of antimicrobial agents, the treatments of methicillin-resistant *S. aureus*-related infections become challenging. For instance, the extensive use of vancomycin led to minimum inhibitory concentration creep, which predominately reduced its clinical efficacy. Due to this action, the emergence of heteroresistant vancomycin-resistant *S. aureus* strains evolved and became resistant to most of the commercial antibiotics. Researchers found that the last-line of linezolid and daptomycin drugs causes adverse side effects and leads to myelosuppression and neurotoxicity (Aljohani et al., 2020; Keikha and Karbalaei, 2024). This underscores the development of new strategies to prevent the methicillin-resistant *S. aureus* (MRSA) infections such as development of vaccine using reverse vaccinology. The recent effort demonstrates the complexity of eliciting protective immunity against disease-causing pathogens using reverse vaccinology. For example, SA4Ag, a vaccine candidate based on capsular polysaccharides, failed to show protective efficacy in clinical trials. This failure was primarily due to the high antigenic variability of capsular polysaccharides among different *Staphylococcus aureus* strains, the immune evasion strategies employed by the bacteria, and the limited capacity of antibody-mediated responses alone to prevent invasive infections (Hassanzadeh et al., 2023; Miller et al., 2020). While they potentially exhibited antibody responses in early trials, they failed in translating immunogenicity into clinical protection. Nevertheless, some studies provided promising preclinical studies; for example, the development of an mRNA-based vaccine for *S. aureus*, such as the staphylococcal enterotoxin B vaccine, provided promising preclinical immunogenicity through experimentation in animal models, thus highlighting the possibility of next-generation vaccines targeting MRSA (Luo et al., 2024).

To target the vaccine candidates, the researchers employed immunoinformatic approaches to select antigenic peptides and subsequent vaccine construction. For instance, multivalent vaccine approaches targeting surface virulence factors, including clumping factor A (clfA), Staphylococcal Enterotoxin B (SEB), alpha-hemolysin, fibronectin binding protein A (FnBPA), and manganese transporter proteins, were evaluated for their potential as vaccine targets (Gao et al., 2025). These findings highlighted the complexity of the protective immunity against *S. aureus*. To address the limitations identified in previous literature, the current study employed a subtractive genomic approach to identify potential vaccine candidates against MRSA. During this target selection. Potential risk of immune cross-reactivity was considered for safety concerns. In spite of that, proteomic analysis against the human proteome using BLASTp (E-value <10^–5^, identity <30%, and coverage greater than 70%) confirmed that the selected protein should be absent in humans, suggesting it reduces the risk of comparative antigenic determinants and minimizes the likelihood of off-target immune recognition.

Through subtractive-genomic analysis, this study identified acetolactate synthase (ALS) or acetohydroxyacid synthase (AHAS) as a novel, essential, non-homologous, and virulent protein. Unlike the classical *S. aureus* candidates such as ClfA and enterotoxins, ALS plays a central role in catalyzing the first step of branched-chain amino acids (BCAA) such as valine, leucine, and isoleucine in the MRSA biosynthesis pathway, which is essential for MRSA’s metabolic functions and virulence (Q. Zhou et al., 2007). Precisely, the research identified that the BCAA biosynthesis pathway is closely linked to MRSA’s metabolic robustness, stress adaptation, and virulence regulation. Nevertheless, this pathway significantly contributes to intercellular pH regulation and redox balance, thereby balancing bacterial persistence under any circumstances (Liang et al., 2025). Thus, targeting BCAA pathway proteins disrupts the intracellular survival, attenuates virulence, and decreases the resistance to oxidative and nitrosative stress conferred within macrophages. This highlights that ALS could potentially serve as an MRSA vaccine candidate and compromise MRSA survival and pathogenicity. Conversely, some of the researchers used subtractive proteomic methodology to identify novel *S. aureus proteins.* Through this analysis, elastin binding protein, glycosyltransferase, and secretory antigen were selected as promising vaccine targets, owing to their essentiality, conservation, and surface accessibility (Tahir ul Tahir ul Qamar et al., 2021). Compared to earlier findings, the present work identified a novel MRSA vaccine target (ALS) using a subtractive genomic approach to elicit robust T-cell-mediated immune responses through MHC class I and II presentation. Thus, it offers a complementary strategy through controlling intracellular *S. aureus* and also addresses the key limitations that have been observed in prior vaccine failures, thereby enhancing the protective immunity.

Constructing multi-target vaccines often provides a broader spectrum of early prevention, minimizes the chances of immune escape, and increases the complexity in epitope selection, antigen processing, structural stability, and manufacturability (Roux et al., 2024). In contrast, this study utilized a single-target vaccine candidate to reduce the variability across strains, thereby enhances the probability of generating a focused and strong immune response that modulates MRSA metabolism. Nevertheless, the reliance on a single antigen also carries potential limitations, including an inadequate immunological breadth and the theoretical risk of strain-specific escape mechanisms (Roux et al., 2024). Thus, combining the ALS vaccine with additional conserved antigens is necessary for developing a multi-target formulation, which further balances immune coverage, safety, and practical feasibility. Most of the researchers employ an integrated immunoinformatic approach along with reverse vaccinology methods to construct a multi-epitope vaccine (MEV). For instance, a study identifies multiple novel antigens, including PPE41 and phospholipase C A from *M. tuberculosis,* and constructs an MEV using convergent methodologies (Sethi et al., 2024). In line with this framework, the present study initially screened B cell and T cell epitopes for ALS targets. These epitope predictions are important for simulating the innate and adaptive immune responses and acquiring long-term immunity. Based on this, the MEV vaccine was constructed with suitable adjuvants and linkers to enhance the immunogenicity (Shahab et al., 2023). To access the vaccine’s potential, the immunological characteristics were analyzed, and the designed ALS vaccine passed the criteria and could potentially be utilized for stimulating immune responses in host organisms.

Further, the population coverage indicated that the designed ALS vaccine covered approximately 98% of the world population, underscoring the translational potential of the selected epitopes. Simultaneously, the docking and simulation studies demonstrated that the ALS vaccine exhibited stable and favourable binding interactions with toll-like receptors 2 and 4. In line with this, MEV constructed for pneumococcal surface protein A (PspA), an important virulence factor in *Streptococcus pneumoniae,* and displayed that the lowest binding energy was observed to be −809.3 kcal/mol with the TLR4 receptor (Shafaghi et al., 2023). In parallel, the recent study that developed a vaccine for tuberculosis revealed that the constructed vaccine (CP91110P), exhibited better binding interactions with TLR4 (−1,672.5 kcal/mol) compared to TLR2 (−1,535.9 kcal/mol) (An et al., 2025). Further, the study identified staphylococcal enterotoxins, alpha hemolysin, and FnBPA using the reverse vaccinology method and declared that the predicted peptide provides promising antigenicity and stability and simultaneously activates Th1 mediated immune responses through effectively binding with TLR receptors (Study, 2024). Moreover, the Staphylococcal Protein-A vaccine developed for combating multi drug-resistant MRSA demonstrated that the predicted vaccine exhibited high affinity and broad theoretical HLA coverage while offering a novel antigenic profile (P. Zhou et al., 2025). These findings suggested that the favourable interaction contributes to stable thermodynamic behaviour between the vaccine and TLR receptors, thereby induces immunogenicity. In contrast, the current study showed that ALS had a high binding affinity towards TLR4, indicating that adjuvant-like motifs incorporated into the ALS vaccine are broadly associated with Th1-mediated immune responses. Thus, activation of TLR4 on antigen-presenting cells enhances the production of IL-12 and INFγ-mediated Th1 differentiation, which are known key factors for cellular immunity against various pathogens (Hu et al., 2024). Nevertheless, the high affinity for TLR4 may contribute to inflammation, as its overactivation causes pathological responses like cytokine storms.

Following that, the codon optimization and *in silico* cloning further confirmed the feasibility of the ALS vaccine expression through generating a final construct of 9,541 base pairs. This evidence suggested that the engineered ALS vaccine is suitable for downstream experimental validation. Simultaneously, the immune simulation studies demonstrated elevated levels of most secondary and tertiary immune responses (IgM and IgG) observed. Some research showcased that the production of IgM and IgG subtypes was consistently observed at elevated levels after repeated immunizations, indicating effective B-cell memory formation (48). For instance, the investigations on SARS-CoV-2 and SARS-CoV-2 Omicron variant vaccines demonstrated elevated IgG, IgM, and INF γ productions over months, suggesting humoral and potential Th1 cell-mediated immune responses (Lan and Ao, 2022; Unninayar et al., 2025). Further, the peptide-based tuberculosis vaccine, such as MP3RT, also exhibited elevated IgG/IgM and cytokine productions in *in vivo* mice models, indicating a robust and balanced immune responses involving both B and T cell-mediated immunity (Cheng et al., 2022). This finding provides empirical support for *in silico* examinations. These outcomes demonstrated that the predicted immune response profile of the ALS vaccine has the capacity to stimulate both humoral and cellular immunity.

Nevertheless, this study establishes ALS as a novel MRSA vaccine target and demonstrates the feasibility of MEV design through computational predictions. However, it is essential to recognize that the current study findings entirely depend on computational predictions, which diminish the biological complexity of immune pathways, antigen processing, or host-specific variability. To address these limitations and to validate the *in silico* findings of the constructed ALS vaccine, cytotoxic T-lymphocyte assay, intracellular cytokine staining, TLR activation reporter assay, dendritic cell maturation assay, lymphocyte proliferation assay, and cytokine profiling are examined, followed by *in vivo* immunization, which further confirms the vaccine’s suitability, safety, and protective efficacy.

## 5 Conclusion

This study utilized integrated subtractive genomic and immunoinformatic approaches to identify and exploit acetolactate synthase (ALS) as a novel MRSA therapeutic target, which was categorized as a non-homologous to human, essential, and virulent MRSA protein. Due to its critical role in the biosynthesis of branched amino acids, it has become an attractive vaccine candidate. The rationally designed ALS vaccine covered a high population worldwide and demonstrated strong binding interactions and stable structural stability with immune receptors. Further, *in silico* profiling also revealed that the ALS-based vaccine is capable of activating humoral and cellular immune responses as indicated by elevated antibody and cytokine productions. Despite that the current ALS vaccine provides strong antigenic properties; it is necessary to evaluate its potential effectiveness both *in vitro* and *in vivo* to further develop the vaccine candidate. Overall, with these findings, a robust multi-epitope vaccine will be constructed with significant translational potential in combating MRSA infections by targeting key metabolic proteins.

## Data Availability

The datasets presented in this study can be found in online repositories. The names of the repository/repositories and accession number(s) can be found in the article/Supplementary Material.

## References

[B1] Aljohani S. Layqah L. Masuadi E. Al B. Baharoon W. Gramish J. (2020). Journal of infection and public health occurrence of vancomycin MIC creep in methicillin resistant isolates in Saudi Arabia. J. Infect. Public Health 13 (10), 1576–1579. 10.1016/j.jiph.2020.07.008 32859551

[B2] Altschul S. F. Madden T. L. Schäffer A. A. Zhang J. Zhang Z. Miller W. (1997). Gapped BLAST and PSI-BLAST: a new generation of protein database search programs. Nucleic Acids Res. 25 (17), 3389–3402. 10.1093/nar/25.17.3389 9254694 PMC146917

[B3] An Y. Ali S. L. Liu Y. Abduldayeva A. Ni R. Li Y. (2025). CP91110P: a computationally designed multi-epitope vaccine candidate for tuberculosis via TLR-2/4 synergistic immunomodulation. Biol. (Basel). 14, 1196. 10.3390/biology14091196 41007341 PMC12467508

[B4] Ayau P. Bardossy A. C. Sánchez-rosenberg G. F. Ortiz R. Moreno D. Hartman P. (2017). International journal of infectious diseases risk factors for 30-Day mortality in patients with methicillin-resistant Staphylococcus aureus bloodstream infections, 61, 3–6. 10.1016/j.ijid.2017.05.010 28533166

[B5] Chen L. Yang J. Yu J. Yao Z. Sun L. Shen Y. (2005). VFDB: a reference database for bacterial virulence factors. Nucleic Acids Res. 33 (DATABASE ISS), 325–328. 10.1093/nar/gki008 15608208 PMC539962

[B6] Cheng P. Xue Y. Wang J. Jia Z. Wang L. Gong W. (2022). Evaluation of the consistence between the results of immunoinformatics predictions and real-world animal experiments of a new tuberculosis vaccine MP3RT. Front. Cell. Infect. Microbiol. 12, 1–17. 10.3389/fcimb.2022.1047306 36405961 PMC9666678

[B7] Clebak K. T. Malone M. A. (2018). Skin infections. Prim. Care - Clin. Office Pract. 45 (3), 433–454. 10.1016/j.pop.2018.05.004 30115333

[B8] Colovos C. Yeates T. O. (1993). Verification of protein structures: patterns of nonbonded atomic interactions. Protein Sci. 2 (9), 1511–1519. 10.1002/pro.5560020916 8401235 PMC2142462

[B9] De Jong N. W. M. Van Kessel K. P. M. Van Strijp J. A. G. (2019). Immune evasion by staphylococcus aureus. Gram-Positive Pathog. 1991 (6), 618–639. 10.1128/9781683670131.ch39 PMC11590434 30927347

[B10] Deurenberg R. H. Stobberingh E. E. (2008). The evolution of Staphylococcus aureus. Infect. Genet. Evol. 8 (6), 747–763. 10.1016/j.meegid.2008.07.007 18718557

[B11] Dimitrov I. Bangov I. Flower D. R. Doytchinova I. (2014). AllerTOP v.2 - a server for *in silico* prediction of allergens. J. Mol. Model. 20 (6), 2278. 10.1007/s00894-014-2278-5 24878803

[B12] Doytchinova I. A. Flower D. R. (2007). VaxiJen: a server for prediction of protective antigens, tumour antigens and subunit vaccines. BMC Bioinforma. 8, 1–7. 10.1186/1471-2105-8-4 17207271 PMC1780059

[B13] Du Z. Su H. Wang W. Ye L. Wei H. Peng Z. (2021). The trRosetta server for fast and accurate protein structure prediction. Nat. Protoc. 16 (12), 5634–5651. 10.1038/s41596-021-00628-9 34759384

[B14] Gao X. Zheng Y. Wang X. Jin J. Liu C. Yang C. (2025). A multivalent mRNA-LNP cocktail vaccine confers superior ef fi cacy against Staphylococcus aureus infection in murine models. NPJ Vaccines 10, 210. 10.1038/s41541-025-01244-4 40998823 PMC12462430

[B15] Garrido-Palazuelos L. I. Almanza-Orduño A. A. Waseem M. Basheer A. Medrano-Félix J. A. Mukthar M. (2024). Immunoinformatic approach for multi-epitope vaccine design against Staphylococcus aureus based on hemolysin proteins. J. Mol. Graph. Model. 132 (July), 108848. 10.1016/j.jmgm.2024.108848 39182254

[B16] Grote A. Hiller K. Scheer M. Münch R. Nörtemann B. Hempel D. C. (2005). JCat: a novel tool to adapt codon usage of a target gene to its potential expression host. Nucleic Acids Res. 33 (Suppl. 2), 526–531. 10.1093/nar/gki376 15980527 PMC1160137

[B17] Gupta S. Kapoor P. Chaudhary K. Gautam A. Kumar R. Raghava G. P. S. (2013). *In silico* approach for predicting toxicity of peptides and proteins. PLoS ONE 8 (9), e73957. 10.1371/journal.pone.0073957 24058508 PMC3772798

[B18] Gupta B. B. Soman K. C. Bhoir L. Gadahire M. Patel B. Ahdal J. (2021). Burd. Methicillin Resist. Staphylococcus aureus Surg. Site Infect. A Rev., 1–6. 10.7860/JCDR/2021/46922.14891

[B19] Hasanpour A. H. Sepidarkish M. Mollalo A. Ardekani A. Almukhtar M. Mechaal A. (2023). The global prevalence of methicillin-resistant Staphylococcus aureus colonization in residents of elderly care centers: a systematic review and meta-analysis. Antimicrob. Resist. Infect. Control 12 (1), 1–11. 10.1186/s13756-023-01210-6 36709300 PMC9884412

[B20] Hassanzadeh H. Baber J. Begier E. Noriega D. C. Konishi H. Yato Y. (2023). Efficacy of a 4-Antigen Staphylococcus aureus vaccine in spinal surgery: the STaphylococcus aureus suRgical inpatient vaccine efficacy (STRIVE) randomized clinical trial. Clin. Infect. Dis. 77 (2), 312–320. 10.1093/cid/ciad218 37125490 PMC10371312

[B21] Hu A. Sun L. Lin H. Liao Y. Yang H. Mao Y. (2024). Harnessing innate immune pathways for therapeutic advancement in cancer. Signal Transduct. Target. Ther. 9, 68. 10.1038/s41392-024-01765-9 38523155 PMC10961329

[B22] Kanehisa M. Furumichi M. Sato Y. Matsuura Y. Ishiguro-Watanabe M. (2025). KEGG: biological systems database as a model of the real world. Nucleic Acids Res. 53 (D1), D672–D677. 10.1093/nar/gkae909 39417505 PMC11701520

[B23] Karim M. Nazrul Islam M. Jewel G. M. N. A. (2020). *In silico* identification of potential drug targets by subtractive genome analysis of Enterococcus faecium DO. BioRxiv. 10.1101/2020.02.14.948232

[B24] Keikha M. Karbalaei M. (2024). Global distribution of heterogeneous vancomycin-intermediate Staphylococcus aureus strains (1997-2021): a systematic review and meta-analysis. J. Glob. Antimicrob. Resist. 37, 11–21. 10.1016/j.jgar.2024.02.002 38336227

[B25] Khan K. Alhar M. S. O. Abbas M. N. Abbas S. Q. Kazi M. Khan S. A. (2022a). Integrated bioinformatics-based subtractive genomics approach to decipher the therapeutic drug target and its possible intervention against brucellosis. Bioengineering 9 (11), 633. 10.3390/bioengineering9110633 36354544 PMC9687753

[B26] Khan K. Jalal K. Khan A. Al-Harrasi A. Uddin R. (2022b). Comparative metabolic pathways analysis and subtractive genomics profiling to prioritize potential drug targets against Streptococcus pneumoniae. Front. Microbiol. 12 (February), 1–16. 10.3389/fmicb.2021.796363 35222301 PMC8866961

[B27] Kozakov D. Hallc D. R. Xiab B. Porterb K. A. Padhornya D. Yuehb C. (2017). 乳鼠心肌提取 HHS public access. ClusPro Web Serv. Protein-Protein Docking 12 (1), 255–278. 10.1038/nprot.2016.169.The 28079879 PMC5540229

[B28] Kumar A. Misra G. Mohandas S. Yadav P. D. (2024). Multi-epitope vaccine design using *in silico* analysis of glycoprotein and nucleocapsid of NIPAH virus. PLoS ONE 19 (5 May), 1–27. 10.1371/journal.pone.0300507 38728300 PMC11086869

[B29] Lan T. Ao D. He X. Liu J. Chen L. Baptista-Hon D. T. (2022). SARS-CoV-2 omicron variant: immune escape and vaccine development. MedComm (2020). 3, 1–17. 10.1002/mco2.126 35317190 PMC8925644

[B30] Laskowski R. A. Hutchinson E. G. Michie A. D. Wallace A. C. Jones M. L. Thornton J. M. (1997). PDBsum: a web-based database of summaries and analyses of all PDB structures. Trends Biochem. Sci. 22 (12), 488–490. 10.1016/S0968-0004(97)01140-7 9433130

[B31] Lemkul J. (2019). From proteins to perturbed hamiltonians: a suite of tutorials for the GROMACS-2018 molecular simulation package. Living J. Comput. Mol. Sci. 1 (1), 0–53. 10.33011/livecoms.1.1.5068

[B32] Liang Y. Niu Z. Wu Z. Zhang Q. Zhao X. Chao L. (2025). Catalytic insights of acetolactate synthases from different bacteria. Archives Biochem. Biophysics 764 (November 2024), 110248. 10.1016/j.abb.2024.110248 39617118

[B33] Luo F. Xu C. Zhang C. Tan A. Lu D. Luo P. (2024). mRNA-based platform for preventing and treating Staphylococcus aureus by targeted staphylococcal enterotoxin B. November, 1–14. 10.3389/fimmu.2024.1490044 PMC11617584 39640268

[B34] Lyon L. M. Marroquin S. M. Thorstenson J. C. Joyce L. R. Fuentes E. J. Doran K. S. (2025). Genome-wide mutagenesis identifies factors involved in MRSA vaginal colonization. Cell Rep. 44 (3), 115421. 10.1016/j.celrep.2025.115421 40085646 PMC12483769

[B35] McGuffin L. J. Bryson K. Jones D. T. (2000). The PSIPRED protein structure prediction server. Bioinformatics 16 (4), 404–405. 10.1093/bioinformatics/16.4.404 10869041

[B36] Miller L. S. Fowler V. G. Shukla S. K. Rose E. Proctor R. A. (2020). Development of a vaccine against Staphylococcus aureus invasive infections: evidence based on human immunity, genetics and bacterial evasion mechanisms pathways implicated in protection evasion mechanisms that counteract. FEMS Microbiol. Rev. 44, 123–153. (Issue September 2019). 10.1093/femsre/fuz030 31841134 PMC7053580

[B37] Munieswaran G. Subramani N. K. Venugopal S. (2025). Machine learning prediction and simulation of drugs targeting GSK-3β in breast cancer. Curr. Drug Ther. 20 (2), 196–209. 10.2174/0115748855333541240819111638

[B38] Naidu S. T. (2023). Marshall digital scholar effects of disulfiram on the metabolome of MRSA.

[B39] Naorem R. S. Pangabam B. D. Bora S. S. Goswami G. Barooah M. Hazarika D. J. (2022). Identification of putative vaccine and drug targets against the Methicillin-resistant Staphylococcus aureus by reverse vaccinology and subtractive genomics approaches. Molecules 27 (7), 2083. 10.3390/molecules27072083 35408485 PMC9000511

[B40] Rapin N. Lund O. Bernaschi M. Castiglione F. (2010). Computational immunology meets bioinformatics: the use of prediction tools for molecular binding in the simulation of the immune system. PLoS ONE 5 (4), e9862. 10.1371/journal.pone.0009862 20419125 PMC2855701

[B41] Rønning T. G. Enger H. Afset J. E. Ås C. G. (2025). Trends and characteristics of multidrug-resistant MRSA in. Front. Microbiol. 16, 1564943. 10.3389/fmicb.2025.1564943 40415932 PMC12098411

[B42] Roux H. Touret F. Rathelot P. Vanelle P. (2024). “From the “ One-Molecule, One-Target, One-Disease ” concept towards looking for multi,” in Target therapeutics for treating non-polio enterovirus (NPEV) infections. 10.3390/ph17091218 PMC11434921 39338380

[B43] Saha S. Raghava G. P. S. (2006). VICMpred: an SVM-based method for the prediction of functional proteins of gram-negative bacteria using amino acid patterns and composition. Genomics, Proteomics Bioinforma. 4 (1), 42–47. 10.1016/S1672-0229(06)60015-6 16689701 PMC5054027

[B44] Samad A. Ahammad F. Nain Z. Alam R. Imon R. R. Hasan M. (2020). Designing a multi-epitope vaccine against SARS-CoV-2: an immunoinformatics approach. J. Biomol. Struct. Dyn. 0 (0), 1–17. 10.1080/07391102.2020.1792347 32677533 PMC7441805

[B45] Sethi G. Varghese R. P. Lakra A. K. Nayak S. S. Krishna R. Hwang J. H. (2024). Immunoinformatics and structural aided approach to develop multi-epitope based subunit vaccine against Mycobacterium tuberculosis. Sci. Rep. 14 (1), 1–16. 10.1038/s41598-024-66858-5 38987613 PMC11237054

[B46] Shafaghi M. Bahadori Z. Madanchi H. Ranjbar M. M. Shabani A. A. Mousavi S. F. (2023). Immunoinformatics - aided design of a new multi - epitope vaccine adjuvanted with domain 4 of pneumolysin against Streptococcus pneumoniae strains. BMC Bioinforma. 24, 1–27. 10.1186/s12859-023-05175-6 36829109 PMC9951839

[B47] Shahab M. Alzahrani A. K. Duan X. Aslam M. Imran M. Kamal M. (2023). An immunoinformatics approach to design novel and potent multi-epitope-based vaccine to target lumpy skin disease. Biomedicines 11 (2), 398. 10.3390/biomedicines11020398

[B48] Study C. (2024). A computational approach to developing a multi-epitope vaccine for combating Pseudomonas aeruginosa – induced. 25(5). 10.1093/bib/bbae401 PMC11318047 39133098

[B49] Subramani N. K. Venugopal S. (2025). Identification of novel drug targets and small molecule discovery for MRSA infections. Front. Bioinforma. 5 (April), 1–16. 10.3389/fbinf.2025.1562596 40303563 PMC12037569

[B50] Szklarczyk D. Kirsch R. Koutrouli M. Nastou K. Mehryary F. Hachilif R. (2023). The STRING database in 2023: protein-protein association networks and functional enrichment analyses for any sequenced genome of interest. Nucleic Acids Res. 51 (1 D), D638–D646. 10.1093/nar/gkac1000 36370105 PMC9825434

[B51] Tahir ul Qamar M. Ahmad S. Fatima I. Ahmad F. Shahid F. Naz A. (2021). Designing multi-epitope vaccine against Staphylococcus aureus by employing subtractive proteomics, reverse vaccinology and immuno-informatics approaches. Comput. Biol. Med. 132 (January), 104389. 10.1016/j.compbiomed.2021.104389 33866250

[B52] Unninayar D. Falcone E. L. Chapdelaine H. Vinh D. C. Top K. A. Derfalvi B. (2025). Humoral and cell-mediated immune responses to COVID-19 vaccines up to 6 months post three-dose primary series in adults with inborn errors of immunity and their breakthrough infections. Front. Immunol. 2 (January), 1–17. 10.3389/fimmu.2024.1501908 39906736 PMC11790575

[B53] Valathoor M. N. Venugopal S. Rajan A. P. (2025). Subtractive genomic approach to uncover novel drug targets in Salmonella typhimurium and computational screening of food-based polyphenols as inhibitors. Front. Bioinform. 5 (December), 1–14. 10.3389/fbinf.2025.1695217 41415625 PMC12710465

[B54] Vita R. Blazeska N. Marrama D. Curation I. Members T. Duesing S. (2025). The Immune epitope database (IEDB): 2024 update, 436–443. 10.1093/nar/gkae1092 PMC11701597 39558162

[B55] Walker J. M. (2005). The Proteomics Protocols Handbook. Digestion. 10.1385/1592598900

[B56] Wallner B. Elofsson A. (2003). Can correct protein models be identified? Protein Sci. 12 (5), 1073–1086. 10.1110/ps.0236803 12717029 PMC2323877

[B57] Wang Z. Feig J. L. Mannschreck D. B. Cohen B. A. Dmitrenko O. Chaplin A. (2022). Antibiotic sensitivity and clinical outcomes in staphylococcal scalded skin syndrome. World J. Pediatr. 23 (24), 222–223. 10.3390/ijms232416086 31626359

[B58] Waterhouse A. Bertoni M. Bienert S. Studer G. Tauriello G. Gumienny R. (2018). SWISS-MODEL: homology modelling of protein structures and complexes. Nucleic Acids Res. 46 (W1), W296–W303. 10.1093/nar/gky427 29788355 PMC6030848

[B59] WHO (2024). “WHO bacterial priority pathogens list, 2024,” in Bacterial pathogens of public health importance to guide research, development and strategies to prevent and control antimicrobial resistance. Available online at: https://www.who.int/publications/i/item/9789240093461.

[B60] Yu N. Y. Wagner J. R. Laird M. R. Melli G. Rey S. Lo R. (2010). PSORTb 3.0: improved protein subcellular localization prediction with refined localization subcategories and predictive capabilities for all prokaryotes. Bioinformatics 26 (13), 1608–1615. 10.1093/bioinformatics/btq249 20472543 PMC2887053

[B61] Zhai Y. Liu L. Zhang F. Chen X. Wang H. Zhou J. (2025). Network pharmacology: a crucial approach in traditional Chinese medicine research. Chin. Med. (United Kingdom) 20 (1), 1–20. 10.1186/s13020-024-01056-z 39800680 PMC11725223

[B62] Zhang R. Ou H. Y. Zhang C. T. (2004). DEG: a database of essential genes. Nucleic Acids Res. 32 (DATABASE ISS), 271–272. 10.1093/nar/gkh024 14681410 PMC308758

[B63] Zhou Q. Liu W. Zhang Y. Liu K. K. (2007). Action mechanisms of acetolactate synthase-inhibiting herbicides. Pesticide Biochem. Physiology 89 (2), 89–96. 10.1016/j.pestbp.2007.04.004

[B64] Zhou P. Shi X. Xia J. Wang Y. Dong S. (2025). Innovative epitopes in staphylococcal Protein-A an immuno-informatics approach to combat MDR-MRSA infections. January 14, 1503944. 10.3389/fcimb.2024.1503944 39877652 PMC11772303

